# Loureirin B Alleviates Myocardial Ischemia/Reperfusion Injury via Inhibiting PAI-1/TGF-*β*1/Smad Signaling Pathway

**DOI:** 10.1155/2022/9128210

**Published:** 2022-04-30

**Authors:** Fei Kong, Meng Zhao

**Affiliations:** ^1^Department of Endocrinology, The Fourth Affiliated Hospital of Heilongjiang University of Traditional Chinese Medicine, Harbin, Heilongjiang, China; ^2^Heilongjiang University of Chinese Medicine, Harbin, Heilongjiang 150040, China; ^3^The First Department of Surgery, First Affiliated Hospital, Heilongjiang University of Chinese Medicine, Harbin, Heilongjiang 150040, China

## Abstract

Myocardial ischemia/reperfusion (MI/R) injury is a common clinical problem after myocardial infarction without effective therapy. Loureirin B (LrB) is a kind of flavonoid with anti-inflammatory and antifibrotic activities. However, the effect of LrB on MI/R and its underlying mechanism remains elusive. In the present study, a mouse model of MI/R was established by coronary artery occlusion. Administration of LrB (0.5 mg/kg or 1 mg/kg) for 4 weeks effectively improved left ventricular (LV) function and reduced myocardial infarction in MI/R mice. MI/R-induced expression of IL-6, TNF-*α*, and IL-1*β* in the hearts was reduced by LrB treatment. Histological analysis showed that LrB attenuated myocardial collagen deposition. LrB downregulated fibronectin, collagen I, collagen III, and *α*-SMA expression. Notably, LrB inhibited the expression of profibrotic plasminogen activator inhibitor-1 (PAI-1), transforming growth factor (TGF)-*β*1, TGF-*β*1R, and p-Smad2/3. Consistently, LrB inhibited the activation of TGF-*β*1/Smad signaling pathway and the expression of fibrosis-related proteins in angiotensin (Ang) II-treated cardiac fibroblasts (CFs). Overexpression of PAI-1 abolished the effects of LrB on Ang II-treated CFs, suggesting that LrB may function through regulating PAI-1. These results indicated that LrB may alleviate MI/R-induced myocardial fibrosis by inhibiting PAI-1/TGF-*β*1/Smad signaling pathway. Thus, LrB may be a potential drug in the treatment of MI/R injury.

## 1. Introduction

Myocardial infarction (MI) is a leading cause of death in patients with cardiovascular diseases [1]. Timely reperfusion by thrombolytic treatment or surgical intervention is considered as the most effective method to reduce infarct size and recover cardiac function after MI [2]. However, the reperfusion after ischemia may aggravate cell death and cause irreversible damages to the myocardial structure [3]. Unfortunately, effective therapy for preventing myocardial ischemia/reperfusion (MI/R) injury has not been developed so far [4]. Therefore, it is still of great importance to understand the molecular mechanism and to seek for effective strategies to prevent MI/R injury. Increasing evidence has uncovered that inflammation is a predominant event involved in the pathogenesis of MI/R injury, which is advanced by various proinflammatory cytokines, such as tumor necrosis factor (TNF)-*α* and interleukin (IL)-1*β* [5, 6]. TNF-*α* has been reported to lead to MI/R-induced adverse left ventricular (LV) remodeling and contractile dysfunction [7]. During the process of pathological LV remodeling, cardiac fibroblasts expand and migrate to the injured lesions and transdifferentiate into myofibroblasts. The myofibroblasts contribute greatly to excessive extracellular matrix (ECM) deposition and fibrotic scar formation, which may eventually result in myocardial fibrosis and heart failure [8, 9].

Sanguis draxonis (also known as “Dragon's Blood”) is a traditional Chinese herbal medicine with a variety of pharmacological activities (anti-inflammation, analgesia, antibiosis, antithrombosis, etc.), which has been clinically applied in the treatment of trauma, gynecopathy, and blood stasis syndrome [10]. A recent report demonstrated that sanguis draxonis can protect against the apoptosis of myocardial cells induced by MI/R in tree shrews [11]. Loureirin B (LrB) is a kind of flavonoid separated from sanguis draxonis. Sun et al. found that LrB can attenuate the inflammation in an experimental inflammatory bowel disease model of rats [12]. Bai et al. reported that LrB inhibits fibroblast proliferation and extracellular matrix deposition in the scar tissue of rabbits [13]. These findings suggest the anti-inflammatory and antifibrotic functions of LrB. Nonetheless, little is known about the role of LrB in MI/R-induced inflammatory response and fibrosis.

Plasminogen activator inhibitor-1 (PAI-1) is a negative regulator in the fibrinolytic system, which inhibits the activity of tissue-type plasminogen activator (tPA) and urokinase-type plasminogen activator (uPA) [14]. Growing evidence has indicated that PAI-1 participates in the pathogenesis of various fibrosis-related diseases, such as liver fibrosis, lung fibrosis, and kidney fibrosis [15–17]. Moreover, plasma AI-1 is upregulated in patients with MI [18]. In a mouse model of MI, PAI-1 is highly expressed in the cardiac tissues and is demonstrated to contribute to myocardial fibrosis [19]. These findings implicate that PAI-1 may play a critical role in MI-induced myocardial fibrosis. Interestingly, LrB has been reported to inhibit the activity of PAI-1 and exhibited potential therapeutic effects on thrombotic diseases and fibrotic diseases [20]. However, the effect of LrB in MI/R injury has not been elucidated yet. In the present study, our aim was to investigate the potential function of LrB in MI/R *in vivo* and *in vitro*. Moreover, the relationship between LrB and PAI-1 was also investigated.

## 2. Materials and Methods

### 2.1. MI/R Model of Mice

All animal studies were approved and performed in accordance with the Ethics Committee of First Affiliated Hospital, Heilongjiang University of Chinese Medicine. Male C57BL/6J mice aged 6 weeks old were adaptively fed for 1 week and divided into sham group, M1/R group, MI/R + 0.5 mg/kg LrB group, and MI/R + 1 mg/kg LrB group. To establish an animal MI/R model, mice were anesthetized by intraperitoneal injection of 50 mg/kg pentobarbital sodium, intubated and mechanically ventilated. After open-chest operation, the left anterior descending coronary artery (LAD) was occluded for 30 min before recovering the blood flow. In the sham group, mice were performed with open-chest operation but did not suffer from MI/R. For drug treatment, mice in the MI/R + 0.5 mg/kg LrB group and the MI/R + 1 mg/kg LrB group received 0.5 mg/kg LrB (Meilunbio, Dalian, China) and 1 mg/kg LrB by intraperitoneal injection per day, respectively [20]. The Sham and MI/R groups were injected with normal saline. Four weeks later, the LV end-diastolic diameter (LVEDD), LV end-systolic diameter (LVESD), LV fractional shortening (LVFS), and LV ejection fraction (LVEF) of the mice were detected using a GE Voluson E8 color Doppler ultrasound diagnosis system (General Electric, Boston, MA, USA).

### 2.2. Determination of Myocardial Infarct Size

Mice were sacrificed by intraperitoneal injection of 200 mg/kg pentobarbital sodium, and the cardiac tissues were collected. After washing with saline, the cardiac tissues were preserved at −20°C for 3 h and then sliced. The slices were immersed in 2 mL 2% TTC solution (Aladdin, Shanghai, China) and incubated at 37°C for 30 min. The infarct area (white or pale) and noninfarct area (red) were analyzed using Image-Pro Plus 6.0 software, and the percentage of infarct area was calculated.

### 2.3. ELISA for Inflammatory Cytokines

Cardiac tissues were mechanically made into 10% homogenate on ice and centrifuged at 430 g for 10 min to obtain the supernatant. Protein concentration was determined by a BCA assay kit (Beyotime, Haimen, China) according to the protocols. Concentrations of TNF-*α*, IL-1*β*, and IL-6 in the supernatant were assessed by commercial ELISA kits following the manufacturer's instructions (USCN Life Science, Wuhan, China).

### 2.4. Masson's Trichrome Staining

The cardiac tissues were successively immersed in 70%, 80%, 90%, and 100% ethanol for dehydration, embedded in paraffin, and sectioned into slices of 5 *μ*m. For Masson's trichrome staining, the slices were incubated with hematoxylin solution (Solarbio, Beijing, China) for 6 min and washed with distilled water. After drying using absorbent paper, the slices were incubated with Ponceau S and acid fuchsin solution (Sinopharm, Beijing, China) for 1 min and then rinsed with 0.2% acetic acid solution. At last, the slices were stained with aniline blue solution and examined under a microscope (OLYMPUS, Tokyo, Japan). The quantitative analysis of the relative staining was performed using Image-Pro Plus 6.0 software.

### 2.5. Immunofluorescence Staining for PAI-1

The cardiac tissue slices were repaired with antigen retrieval solution for 10 min and rinsed with phosphate buffer solution (PBS) for 3 times. After immersion in goat serum (Solarbio) at room temperature for 15 min, the slices were incubated with primary antibody for PAI-1 (1 : 100 dilution; Abcam, Cambridge, UK) at 4°C overnight and rinsed with PBS for 3 times. Then, the slices were incubated with Cy3-labeled goat anti-rabbit immunoglobulin G (IgG) (1 : 200 dilution; Beyotime) at room temperature for 60 min. At last, the slices were stained with DAPI solution (Beyotime). The developed tissue slices were mounted and examined under a fluorescence microscope.

### 2.6. Isolation of Cardiac Fibroblasts (CFs)

The hearts of mice were harvested in a sterile environment and infused with collagenase II-contained perfusion buffer solution for 3 min. Then, the heart was perfused with digestive buffer solution supplemented with 12.5 *μ*M CaCl_2_ for 8 min. After cutting into small pieces, the heart tissues were treated with buffer solution supplemented with 12.5 *μ*M CaCl_2_ and 10% fetal bovine serum (Hyclone, Logan, UT, USA), filtered to remove undigested tissues, and rested for 20 min to collect the cells. The isolated cells were resuspended in the perfusion buffer solution containing 12.5 *μ*M CaCl_2_ and 5% fetal bovine serum and rested for 20 min to collect the supernatant. After centrifugation at 500 g for 7 min, the cells were seeded onto culture plates coated with 1% gelatin and incubated in Iscove's modified Dulbecco's medium (IMDM; Procell, Wuhan, China) at 37°C.

### 2.7. Cell Identification

For identification, the cells were fixed in 4% paraformaldehyde, permeabilized with 0.1% Triton X-100 (Beyotime) for 30 min, and rinsed with PBS for 3 times. After treatment with goat serum for 15 min, the cells were incubated with primary antibody against fibroblast-specific protein 1 (FSP1) (1 : 200 dilution; ABclonal, Wuhan, China) or *α*-smooth muscle actin (*α*-SMA) (1 : 200 dilution; Abcam) at 4°C overnight. The cells were rinsed with PBS for 3 times and then incubated with Cy3-labeled goat anti-rabbit IgG (1 : 200 dilution; Beyotime) at room temperature for 60 min. At last, the cells were stained by DAPI solution and observed under a microscope.

### 2.8. Cell Treatment

CFs were divided into control group, angiotensin (Ang) II group, Ang II + LrB group, Ang II + LrB + vector group, and Ang II + LrB + PAI-1 OE group. Cells in the Ang II + LrB + PAI-1 OE group were transfected with lentivirus-mediated PAI-1 overexpression (OE) vector, while cells in the Ang II + LrB + vector group were transfected with empty vector. After 48 h, cells in LrB treatment groups were incubated with 25 *μ*g/mL LrB [21] for 1 h. Then, cells in angiotensin (Ang) II treatment groups were incubated with 0.1 *μ*M Ang II (GL Biochem, Shanghai, China) for 24 h.

### 2.9. Western Blot Analysis

The relative protein expression in the cardiac tissues or CFs was analyzed by Western blot. Samples were treated with RIPA lysis buffer (Solarbio) and centrifuged at 10000 g (4°C) for 5 min to obtain the protein samples. After quantification with the BCA assay kit, the protein samples were separated using SDS-PAGE (8%, 10%, and 15% separation gel) and transferred onto PVDF membranes (Millipore, Billerica, MA, USA). The membranes were immersed in 5% nonfat milk to block the nonspecific binding for 1 h and incubated at 4°C overnight with primary antibody for fibronectin (1 : 2000 dilution; ABclonal), collagen I (1 : 500 dilution; Affinity, Changzhou, China), collagen III (1 : 1000 dilution; Affinity), *α*-SMA (1 : 500 dilution; ABclonal), PAI-1 (1 : 1000 dilution; Abcam), uPA (1 : 500 dilution; Affinity), tPA (1 : 500 dilution; Affinity), TGF-*β*1 (1 : 400 dilution; Affinity), TGF-*β*1R (1 : 1000 dilution; Affinity), p-Smad2/3 (1 : 500 dilution; ABclonal), Smad2/3 (1 : 1000 dilution; ABclonal), and GAPDH (1 : 10000 dilution; Proteintech, Wuhan, China). After rinsing with TBSF buffer, the membranes were incubated with HRP-conjugated goat anti-mouse or goat anti-rabbit IgG (1 : 3000 dilution; Solarbio) at 37°C for 1 h. For visualization of the protein bands, PVDF membranes were treated with ECL reagent (Solarbio) and exposed in the darkroom. The optical density of the protein bands was analyzed using Gel-Pro Analyzer software.

### 2.10. Real-Time PCR

The relative mRNA expression of PAI-1, uPA, and tPA was analyzed by real-time PCR. Total RNA was extracted and quantified (UV spectrophotometer). RNA samples were reverse-transcribed into cDNA templates on a PCR system (BIONEER, Daejeon, Korea). Quantitative real-time PCR was conducted using the template, primers, Taq HS Perfect Mix (TaKaRa, Tokyo, Japan), and SYBR Green (BioTeke, Beijing, China) on the PCR system. The sequences of primers were shown as follows: PAI-1F, 5′-ATGCCATCTTTGTCCAGC-3′; PAI-1R, 5′-TCTGAGAAGTCCACCTGTTT-3′; uPA F, 5′-TAGACCAACAAGGCTTCC-3′; uPA R, 5′-GAGACTCCCACCACATTTA-3′; tPA F, 5′-AGTGCCCTGATGGATTTG-3′; tPA R, 5′-GTCTCGGTCTGGGTTTCT-3′.

### 2.11. Statistical Analysis

All data were shown as mean values ± SD. Statistical difference was analyzed by one-way analysis of variance using GraphPad Prism 8.0 software, followed by Turkey's test for multiple comparison. *p* < 0.05 was considered statistically significant.

## 3. Results

### 3.1. LrB Improved Cardiac Function and Reduced Infarct Size in MI/R Mice

**Figure 1:**
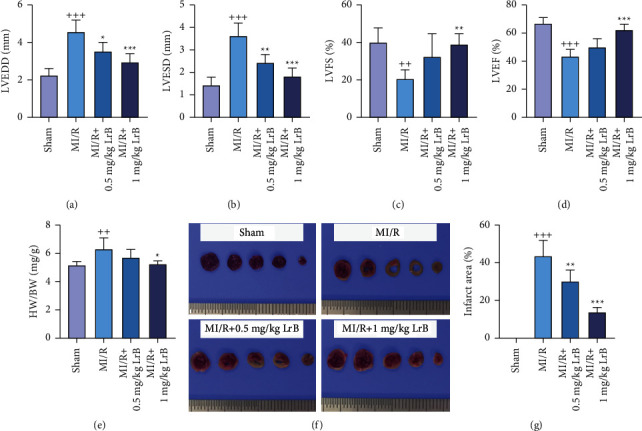
Loureirin B (LrB) improved cardiac function and reduced infarct size in mice with myocardial ischemia/reperfusion (MI/R). Mice were subjected to MI/R and treated with 0.5 mg/kg LrB or 1 mg/kg LrB per day for 4 weeks. Four weeks later, cardiac function of the mice was measured, and the cardiac tissues were harvested for further examination. (a) Left ventricular end-diastolic diameter (LVEDD), (b) left ventricular end-systolic diameter (LVESD), (c) left ventricular fractional shortening (LVFS), and (d) left ventricular ejection fraction (LVEF) measured by echocardiographic examination. (e) The ratio of heart weight (HW) to body weight (BW). (f) Representative photographs of heart cross sections after TTC staining. (g) The percentage of myocardial infarct area. Values are expressed as mean ± SD (*n* = 6). ^++^*p* < 0.01, ^+++^*p* < 0.001*vs.* sham group. ^*∗*^*p* < 0.05, ^*∗∗*^*p* < 0.01, ^*∗∗∗*^*p* < 0.001*vs.* MI/R group.

Four weeks after MI/R, cardiac function of mice was evaluated by echocardiographic examination. It was found that mice in the MI/R group showed significantly higher LVEDD and LVESD than mice in the sham group (Figures 1(a) and 1(b)). Moreover, MI/R mice exhibited significantly lower LVFS and LVEF than mice in the sham group (Figures 1(c) and 1(d)), indicating that MI/R resulted in a decline in LV function in mice. Treatment with 0.5 mg/kg or 1 mg/kg LrB decreased LVEDD and LVESD in MI/R mice. However, only 1 mg/kg LrB effectively increased LVFS and LVEF in MI/R mice, demonstrating that LrB might improve the cardiac function of MI/R mice in a dose-dependent manner. Additionally, the ratio of heart weight (HW) to body weight (BW) in MI/R mice was decreased by treatment with 1 mg/kg LrB (Figure 1(e)). TTC staining was then performed to access the infarct size. As shown in Figure 1(f), heart cross sections of MI/R mice showed large white infarct area, while the sham group showed normal red color. Treatment with 0.5 mg/kg or 1 mg/kg LrB reduced infarct size in MI/R mice (Figures 1(f) and 1(g)), demonstrating the beneficial effect of LrB on alleviating MI/R injury.

### 3.2. LrB Attenuated MI/R-Induced Inflammation in Mice

**Figure 2:**
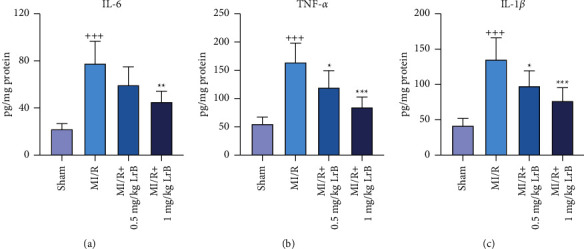
LrB attenuated MI/R-induced inflammation in mice. The concentrations of (a) interleukin (IL)-6, (b) tumor necrosis factor (TNF)-*α*, and (c) IL-1*β* in the cardiac tissues of the mice. Values are expressed as mean ± SD (*n* = 6). ^+++^*p* < 0.001*vs.* sham group. ^*∗*^*p* < 0.05, ^*∗∗*^*p* < 0.01, ^*∗∗∗*^*p* < 0.001*vs.* MI/R group.

The levels of IL-6, TNF-*α*, and IL-1*β* in the cardiac tissues were determined to evaluate the effect of LrB on MI/R-induced inflammation. We found that mice in the MI/R group showed dramatically enhanced levels of IL-6, TNF-*α*, and IL-1*β* compared with the sham group (Figures 2(a)–2(c)), indicating that MI/R caused severe inflammation in the cardiac tissues of mice. Treatment with LrB attenuated the levels of these inflammatory cytokines in a dose-dependent manner, demonstrating the anti-inflammatory effect of LrB on MI/R mice.

### 3.3. LrB Alleviated MI/R-Induced Myocardial Fibrosis in Mice

**Figure 3:**
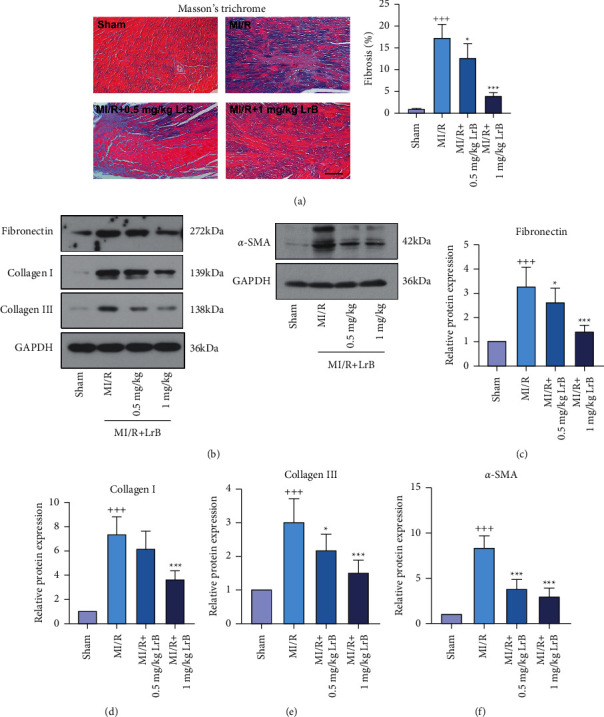
LrB alleviated MI/R-induced myocardial fibrosis in mice. (a) Representative images of the cardiac tissues after Masson's trichrome staining and the percentage of fibrosis were analyzed. Scar bar = 100 *μ*m. (b) Western blot analysis for fibronectin, collagen I, collagen III, and *α*-SMA in the cardiac tissues. Relative protein expression of (c) fibronectin, (d) collagen I, (e) collagen III, and (f) *α*-SMA. Values are expressed as mean ± SD (*n* = 6). ^+++^*p* < 0.001*vs.* sham group. ^*∗*^*p* < 0.05, ^*∗∗∗*^*p* < 0.001*vs.* MI/R group.

MI/R-induced myocardial fibrosis was assessed by Masson's trichrome staining. As illustrated in Figure 3(a), the MI/R group exhibited noticeably increased deposition of collagen fiber in the cardiac tissues compared with the sham group. Notably, treating with 0.5 mg/kg or 1 mg/kg LrB effectively reduced the deposition of collagen fiber induced by MI/R. To verify this observation, the expression of fibrosis-associated proteins fibronectin, collagen I, collagen III, and *α*-SMA in the cardiac tissues was determined by Western blot analysis. As shown in Figures 3(b)–3(f), mice in the MI/R group expressed significantly higher fibronectin, collagen I, collagen III, and *α*-SMA, which were abolished after LrB treatment. These results suggested that LrB could alleviate MI/R-induced myocardial fibrosis in mice.

### 3.4. LrB Suppressed the Expression of PAI-1 and the Activation of TGF-*β*1/Smad Signaling Pathway in MI/R Mice

**Figure 4:**
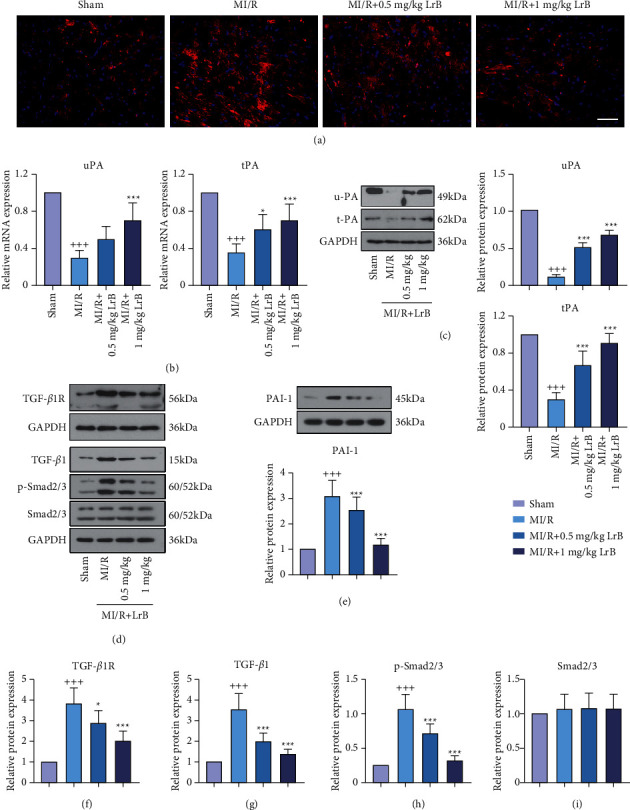
LrB suppressed the expression of PAI-1 and the activation of TGF-*β*1/Smad signaling pathway in MI/R mice. (a) Representative images showing the distribution of PAI-1 in cardiac tissues after immunofluorescence staining. Scar bar = 50 *μ*m. (b, c) Relative mRNA and protein expression of urokinase plasminogen activator (uPA) and tissue-type plasminogen activator (tPA) in the cardiac tissues. (d) Western blot analysis for TGF-*β*1R, TGF-*β*1, p-Smad2/3, and Smad2/3. Relative protein expression of (e) PAI-1, (f) TGF-*β*1R, (g) TGF-*β*1, (h) p-Smad2/3, and (i) Smad2/3 in the cardiac tissues. Values are expressed as mean ± SD (*n* = 6). ^+++^*p* < 0.001*vs.* sham group. ^*∗*^*p* < 0.05, ^*∗∗*^*p* < 0.01, ^*∗∗∗*^*p* < 0.001*vs.* MI/R group.

The distribution of PAI-1 in cardiac tissues was visualized by immunofluorescence staining. Compared with the sham group, the MI/R group showed more red fluorescence of PAI-1 (Figure 4(a)). By contrast, treating with 1 mg/kg LrB inhibited the expression of PAI-1. In addition, mRNA and protein expression levels of uPA and tPA were dramatically downregulated in the MI/R group, which were reversed by treating with LrB (Figures 4(b) and 4(c)). Furthermore, the protein expression of PAI-1, TGF-*β*1, TGF-*β*1R, p-Smad2/3, and Smad2/3 were determined by Western blot analysis (Figures 4(d)–4(i)). The result confirmed that PAI-1 was significantly upregulated in the MI/R group but dramatically downregulated in the MI/R + 1 mg/kg LrB group. Interestingly, MI/R induced the upregulation of TGF-*β*1, TGF-*β*1R, and p-Smad2/3 in the cardiac tissues, suggesting that the TGF-*β*1/Smad signaling pathway was activated in MI/R mice. Treating with LrB significantly downregulated the expression of TGF-*β*1, TGF-*β*1R, and p-Smad2/3. The expression of Smad2/3 had no significant difference between these groups.

### 3.5. LrB Suppressed Ang II-Induced Activation of TGF-*β*1/Smad Signaling Pathway in CFs via Regulating PAI-1

**Figure 5:**
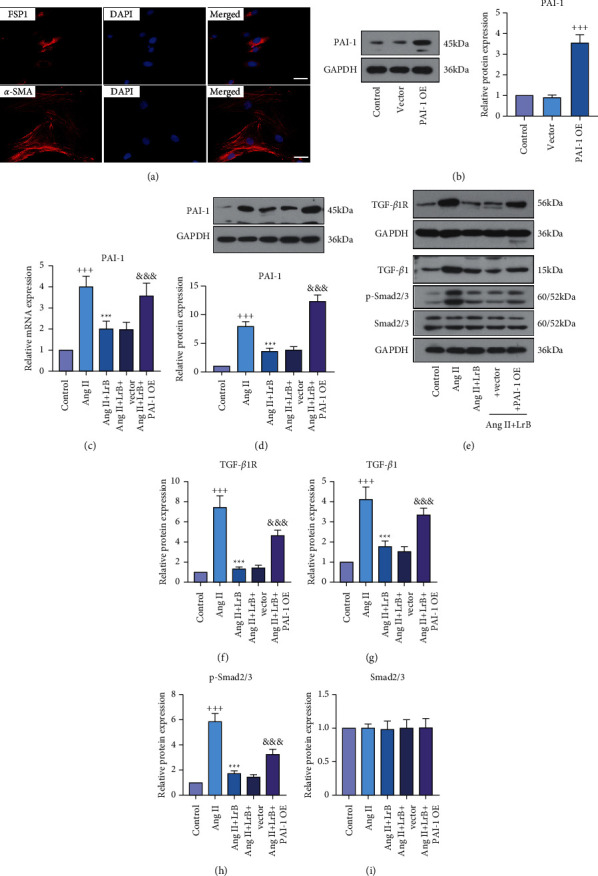
LrB suppressed angiotensin (Ang) II-induced activation of TGF-*β*1/Smad signaling pathway in cardiac fibroblasts (CFs) via regulating PAI-1. (a) Representative fluorescent images of fibroblast-specific protein-1 (FSP1) and *α*-smooth muscle actin (*α*-SMA) in CFs. Scar bar = 50 *μ*m. (b) Western blot analysis for PAI-1 in CFs transfected with empty vector or PAI-1 overexpression (OE) vector. (c, d) Relative mRNA and protein expression of PAI-1 in CFs. (e) Western blot analysis for TGF-*β*1R, TGF-*β*1, p-Smad2/3, and Smad2/3. Relative protein expression of (f) TGF-*β*1R, (g) TGF-*β*1, (h) p-Smad2/3, and (i) Smad2/3 in CFs. Values are indicated as mean ± SD (*n* = 3). ^+++^*p* < 0.001*vs.* control group. ^*∗∗∗*^*p* < 0.001*vs.* Ang II group. ^&&&^*p* < 0.001*vs*. Ang II + LrB + vector group.

FSP1 and *α*-SMA are regarded as specific markers for fibroblasts and myofibroblasts, respectively [22]. Immunofluorescence staining revealed FSP1- and *α*-SMA-positive areas in these cells (Figure 5(a)). Then, the isolated cells were transfected with PAI-1 OE vector to upregulate the expression of PAI-1, as demonstrated by Western blot analysis (Figure 5(b)). Further, we found that 24-h Ang II treatment significantly enhanced the mRNA and protein levels of PAI-1 in CFs (Figures 5(c) and 5(d)). Treating with LrB suppressed the Ang II-induced upregulation of PAI-1, which was reversed by transfection of PAI-1 OE vector. Moreover, Ang II enhanced the protein expression of TGF-*β*1, TGF-*β*1R, and p-Smad2/3 in CFs, which was attenuated by LrB treatment (Figures 5(e)–5(h)). The protein expression of Smad2/3 had no significant difference between these groups (Figure 5(i)). These results indicated that LrB could inhibit the Ang II-induced activation of TGF-*β*1/Smad signaling pathway. Interestingly, transfecting with PAI-1 OE vector greatly enhanced their expression levels, indicating that the upregulation of PAI-1 impaired the effect of LrB in Ang II-treated CFs. Thus, it was speculated that LrB might suppress the Ang II-induced activation of TGF-*β*1/Smad signaling pathway in CFs via regulating PAI-1.

### 3.6. LrB Inhibited Ang II-Induced Expression of Fibronectin, Collagen I, Collagen III, and *α*-SMA in CFs via Regulating PAI-1

**Figure 6:**
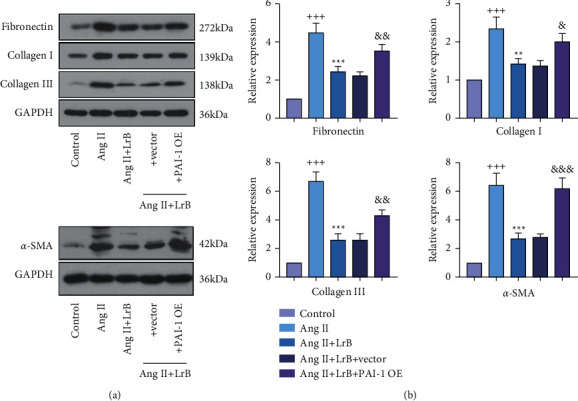
LrB inhibited Ang II-induced synthesis of fibronectin and collagen in CFs via regulating PAI-1. (a) Western blot analysis for fibronectin, collagen I, collagen III, and *α*-SMA in CFs. (b) Relative protein expression of fibronectin, collagen I, collagen III, and *α*-SMA in CFs. Values are indicated as mean ± SD (*n* = 3). ^+++^*p* < 0.001*vs.* control group. ^*∗∗*^*p* < 0.01, ^*∗∗∗*^*p* < 0.001*vs.* Ang II group. ^&^*p* < 0.05, ^&&^*p* < 0.01*vs*. Ang II + LrB + vector group.

We further analyzed the protein expression of fibronectin, collagen I, collagen III, and *α*-SMA in Ang II-treated CFs. It was found that these ECM-related proteins were significantly upregulated in the Ang II group compared with the control group (Figures 6(a) and 6(b)). LrB treatment remarkably attenuated the expression of these ECM-related proteins. Interestingly, transfecting with PAI-1 OE vector upregulated their expression in CFs treated with Ang II and LrB.

## 4. Discussion

MI/R injury can cause arrhythmias, myocardial stunning, microvascular obstruction, and cardiomyocyte damage [2]. Recently, many flavonoids have been demonstrated to show therapeutic effects on MI/R injury. Hu et al. found that luteolin attenuates MI/R injury in mice through regulating calcium transport-associated molecular pathway [23]. Xue et al. reported that vitexin protects against MI/R injury in rats via regulating mitochondrial function [24]. LrB, a kind of flavonoid, has been reported to inhibit oxidative stress in osteoblasts and enhance mitochondrial function after spinal cord injury [25, 26]. However, the potential effect of LrB on MI/R injury has not been reported yet. In the present research, we demonstrated that subjecting to MI/R damaged the LV function, as characterized by decreased LVFS and LVEF. Moreover, MI/R mice suffered massive myocardial infarction, indicating that MI/R induced severe cardiac injury. Interestingly, treating with LrB effectively improved LV function and reduced myocardial infarction size in MI/R mice, suggesting a cardioprotective effect of LrB on MI/R injury.

The mechanism of MI/R damage is complex, and inflammation is the critical pathological feature of cardiac injury [27]. Overactive inflammatory response has been revealed to mediate myocardial cell injury, cardiac dysfunction, and fibrosis [28–32]. To investigate the effect of LrB on MI/R-induced inflammatory response, we determined the concentration of IL-6, TNF-*α*, and IL-1*β* in cardiac tissues. Consistent with previous research, MI/R mice shows significantly increased levels of these proinflammatory cytokines, confirming that MI/R exacerbated inflammation in mice. LrB treatment reduced their levels, demonstrating an anti-inflammatory role of LrB in MI/R. Therefore, we speculated that the anti-inflammatory effect of LrB might be related to its cardioprotective effect on MI/R injury.

LV remodeling refers to a series of compensatory alternations in LV architecture and function in response to MI-induced cardiac injury, which usually results in dilatation, hypertrophy, and deterioration of contractile function [9]. MI/R-induced cardiomyocyte death may provoke adverse LV remodeling, myocardial fibrosis, and heart failure [33]. The pathological alternations in LV remodeling are mediated by the interaction of cardiomyocyte hypertrophy, apoptosis, fibroblasts proliferation, and interstitial fibrosis [34]. During LV remodeling, fibroblasts transform into myofibroblasts and migrate to the injured lesions, resulting in excessive deposition of collagen and fibrosis [35]. In the present study, excessive collagen deposition and upregulation of fibrosis-related proteins (fibronectin, collagen I, collagen III, and *α*-SMA) were detected in the cardiac tissues of MI/R mice. However, treatment with LrB attenuated the collagen deposition and the expression of these fibrosis-related proteins, confirming its potential antifibrotic effect on MI/R injury.

PAI-1, an inhibitor of collagen degradation, plays a pivotal role in MI/R development [36]. To further explore how LrB protected against MI/R-induced myocardial fibrosis, we determined the expression of PAI-1 in the cardiac tissues. PAI-1 was abnormally upregulated in an MI mouse model and identified to induce MI-induced myocardial fibrosis [19]. Moreover, LrB was reported to be an inhibitor of PAI-1 [20]. However, it is unknown whether LrB could regulate the expression of PAI-1 in MI/R mice. In this study, the expression of PAI-1 was found to be dramatically upregulated in untreated MI/R mice, which was in line with previous studies. Treatment with LrB reduced the upregulation of PAI-1, demonstrating that LrB could inhibit PAI-1 expression in MI/R. Therefore, inhibition of PAI might contribute to the antifibrotic effect of LrB in MI/R. However, it should be noted that although high expression of PAI-1 was demonstrated to be profibrotic in the cardiac tissue, PAI-1 is cardioprotective in normal physiological levels, and the deficiency of PAI-1 may also lead to myocardial fibrosis [37, 38]. Therefore, the complex mechanism of LrB's cardioprotective role in MI/R requires further investigation.

TGF-*β*1/Smads is an essential fibrogenic growth signal in myocardial fibrosis induced by MI [39]. It is well described that TGF-*β*1 mediates the activation of Smad2/3 and then activated Smad2/3 binds with Smad4 to form a complex to translocate into the nucleus and regulates specific genes [40]. Also, TGF-*β*1 plays a key profibrotic role in multiple fibrosis-related diseases via regulating fibroblast activation and ECM synthesis [41, 42]. Overexpression of TGF-*β*1 induced myocardial fibrosis in transgenic mice [43]. Blocking TGF-*β*1 signaling alleviated myocardial fibrosis and dysfunction in pressure-overloaded rats [44], implicating the specific role of TGF-*β*1 in promoting myocardial fibrosis. In this study, we found that LrB treatment suppressed the MI/R-induced upregulation of TGF-*β*1, TGF-*β*1R, and p-Smad2/3, revealing that LrB might attenuate MI/R-induced myocardial fibrosis via TGF-*β*1/Smad inactivation, which was correlated with the report of Bai et al. [13].

Activated cardiac fibroblasts play a predominant role in MI/R-induced fibrosis [45]. Ang II has been recognized to enhance myocardial fibrosis through promoting CF activation and production of ECM-related proteins [46]. Here, we found that exposure to Ang II induced the upregulation of PAI-1 and the activation of TGF-*β*1/Smad signaling pathway in CFs, whereas LrB treatment suppressed it. LrB also suppressed the Ang II-induced upregulation of fibronectin, collagen I, collagen III and *α*-SMA in CFs, which could be abolished by overexpressing PAI-1. Therefore, we speculated that LrB might function through regulating PAI-1. In some previous studies, PAI-1 was recognized as a downstream target of TGF-*β*1, which functioned to inhibit ECM degradation and promote organ fibrosis [47, 48]. Other studies suggested that PAI-1 also modulate the activity of TGF-*β*1 in turn [49, 50]. Thus, PAI-1 and TGF-*β*1 may constitute a positive feedback loop and contribute together to the pathogenesis of cardiac fibrosis.

## 5. Conclusions

Collectively, our *in vivo* study showed that treatment with LrB improved LV function and reduced myocardial infarction in MI/R mice, demonstrating a cardioprotective effect of LrB on MI/R injury. Moreover, LrB alleviated MI/R-induced myocardial fibrosis, possibly by inhibiting PAI-1 and TGF-*β*1/Smad. *In vitro* study further demonstrated that LrB reduced the Ang II-induced activation of TGF-*β*1/Smad signaling pathway and production of ECM-related proteins in CFs via regulating PAI-1. These findings support this point that LrB might be an effective therapeutic drug for MI/R injury.

## Data Availability

The data that support the findings of this study are included in the manuscript.

## Supplementary Materials

Supplementary Materials
*Graphical Abstract of the Research*. Loureirin B may alleviate MI/R-induced myocardial inflammatory response and fibrosis through inhibiting PAI-1/TGF-*β*1/Smad signaling pathway.
