# Supplementary material for: Loureirin B Alleviates Myocardial Ischemia/Reperfusion Injury via Inhibiting PAI-1/TGF-β1/Smad Signaling Pathway
Source: Evid Based Complement Alternat Med. 2022 Apr 30;2022:9128210. doi: 10.1155/2022/9128210 (PMC9078770; doi:10.1155/2022/9128210)
Supplement: Supplementary Materials — Graphical Abstract of the Research. Loureirin B may alleviate MI/R-induced myocardial inflammatory response and fibrosis through inhibiting PAI-1/TGF-β1/Smad signaling pathway. [file 9128210.f1.pdf]

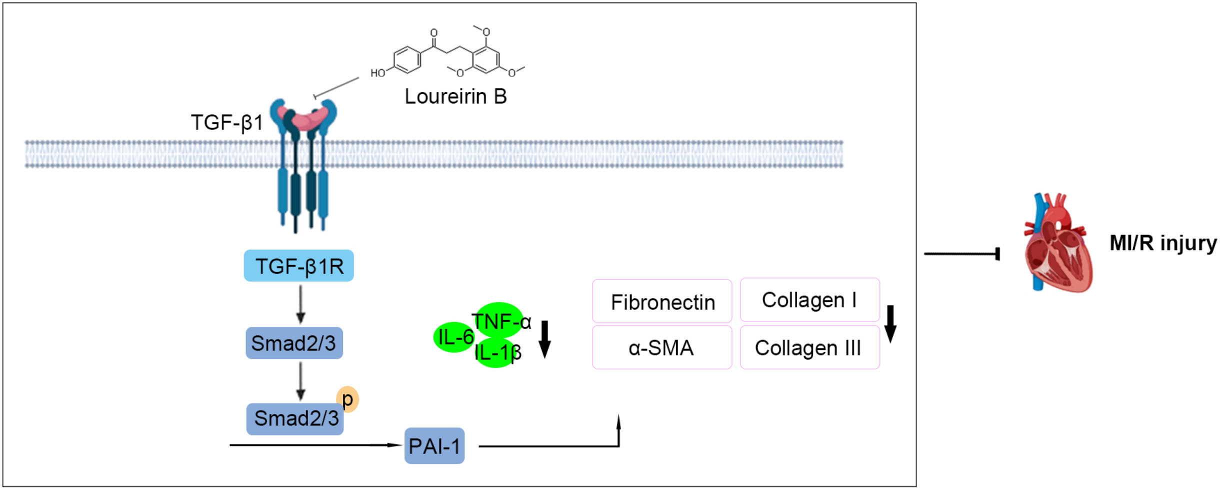

HO
O
Loureirin B
TGF-β1
TGF-β1R
Smad2/3
p
Smad2/3
PAI-1
IL-6
TNF-α
IL-1β
Fibronectin
Collagen I
α-SMA
Collagen III
MI/R injury
